# Understanding the Effect of Electronic Prehospital Medical Records in Ambulances: A Qualitative Observational Study in a Prehospital Setting

**DOI:** 10.3390/ijerph18052330

**Published:** 2021-02-27

**Authors:** Frederikke Bøgh Jensen, Kathrine Tornbjerg Ladefoged, Tim Alex Lindskou, Morten Breinholt Søvsø, Erika Frischknecht Christensen, Maurizio Teli

**Affiliations:** 1 Techno-Anthropology, Technical Faculty of IT and Design, Aalborg University, 9000 Aalborg, Denmark; kathrinetornbjergladefoged@hotmail.com; 2 Centre for Prehospital and Emergency Research, Department of Clinical Medicine, Aalborg University, 9000 Aalborg, Denmark; tim.l@rn.dk (T.A.L.); morten.soevsoe@rn.dk (M.B.S.); efc@rn.dk (E.F.C.); 3 Department of Planning, Aalborg University, 9000 Aalborg, Denmark; maurizio@plan.aau.dk

**Keywords:** ambulances, electronic health record, cultural anthropology, emergency medical services

## Abstract

Little is known of ambulance professionals’ work practices regarding the use of medical records, their communication with patients, before and during hand over to Emergency Departments (ED). An electronic Prehospital Medical Record (ePMR) has been implemented in all Danish ambulances since 2015. Our aim was to investigate the use of ePMR and whether it affected the ambulance professionals’ clinical practice. We performed a qualitative study with observations of ePMR use in ambulance runs in the North Denmark Region. Furthermore, informal interviews with ambulance professionals was performed. Analysis was accomplished with inspiration from grounded theory. Our main findings were: (1) the ePMR is an essential work tool which aided ambulance professionals with overview of data collection and facilitated a checklist for ED hand overs, (2) mobility and flexibility of the ePMR facilitated conversations and relations with the patients, and (3) in acute severe situations, the ePMR could not stand alone in hand over or communication with the ED. The ePMR affected the ambulance professionals’ work practice in various ways and utilization of ePMR while simultaneously treating patients in ambulances does not obstruct the relation with the patient. To this end, the ePMR appears feasible in collaboration across the prehospital setting.

## 1. Introduction

Electronic Patient Records (EPR) have been an object of research since the 1980s [1]. Studies of paper records and EPRs have investigated the positions of EPRs within work practices from a process- and data centered perspective [2,3]. Multiple studies have been performed concerning ‘computer-on-wheels’ and mobile tablet-based documentation in-hospital and found significant benefits for using tablet-based documentation in work practice, e.g., the documentation was performed immediately after the resuscitation, final documentation time was reduced by 34%, and the touch interaction made it easier to navigate in the record [4,5]. Previous studies have furthermore found that the EPRs on mobile tablets introduce flexibility in the mobility work [3]. It can allow health care actors to access timely patient history and provide simultaneously information sharing, which is important when working in different locations, e.g., as ambulance professionals do [3,6]. Written clinical documentation has been identified as important supplementation to verbal hand over in the prehospital setting. Owen et al. found how interruptions often occur when ambulance professionals hand over a patient in the emergency department (ED), how handover requires standardization, and a need for a high level of shared understanding/language between ambulance professionals and ED clinicians [7]. 

EPRs in the prehospital field is a newer innovation and the literature on this subject is sparse. Among others, Australia, Denmark, and some UK ambulance services currently use an EPR in the ambulances [8,9,10]. In Denmark, the nationwide electronic Prehospital Medical Record (ePMR) was implemented in 2015. Prior to this, with the exception of the North Denmark Region, the only available electronic data concerned ambulance dispatch and arrival times, etc., whereas the patient data was noted on paper medical records [11]. The ePMR enables identical data registration in the entire country, where ambulance professionals can enter patient information and measurements, and actors in the prehospital setting, e.g., ED clinicians, can access the ePMR and read information before the ambulance arrives [9,12,13]. In relation to the above-mentioned studies, we find it of great relevance to study how tablet-based documentation affects an out-of-hospital setting, i.e., the prehospital setting, and the hand over from ambulance professionals to ED clinicians, as it can provide important knowledge on the work practice, communication and collaboration amongst the actors in prehospital setting in a Danish context. 

Our aim was to investigate the interaction between ambulance professionals and the ePMR, and to identify how the ePMR affects the ambulance professionals’ work practice.

## 2. Materials and Methods

### 2.1. Study Design

We conducted an ethnographic study of ambulance professionals’ interaction with the ePMR. An ethnographic approach enables an iterative process through which the researcher is able to adapt focus according to how reality unfolds [14]. This was particularly helpful in our study since our research site (i.e., ambulance professionals responding to emergency situations) was highly dynamic and unpredictable. Furthermore, participatory observations were performed with a team ethnographic approach [15,16]. This enabled us to reflect upon phenomena observed on the research site as well as to study how the ambulance professionals interacted and used the ePMR within their natural setting whereby the mundane and taken-for-granted activities and behavior related to using the ePMR were captured. Such activities and behavior are difficult to investigate through other methods since they often are non-reflective making them invisible in interviews, documents (e.g., instructions), etc.

### 2.2. Setting

The Danish Emergency Medical Services (EMS) include ambulances, supplemented with rapid response vehicles as first responders, and mobile emergency care units and helicopter emergency medical services with prehospital doctors [9]. The prehospital doctors were present by the patient on scene in life-threatening situations, and in other situations available for giving advice. Since 2006, the ambulances in the North Denmark Region had the first version of the ePMR and since 2015, all Danish ambulances have used the same ePMR, located on tablet computers. The ePMRs in the ambulances are connected wirelessly to a server for data synchronization. However, they can also function without any real-time or direct connection to the server. Therefore, they can withstand losing the connection and log the data when later regaining it. In the ED, a central ePMR is placed on a counter that allows a coordinating nurse and other health care professionals in the ED to access ePMR data collected in ambulances. The main function of the coordinating nurse is to receive handover regarding the patient from the ambulance professionals, to delegate patients into rooms, and keep the overview in ED.

In Denmark, two levels of ambulance professionals are available with increasing competencies; paramedic and paramedic with special competencies. Ambulances are always manned by at least two ambulance professionals.

### 2.3. Participants

We included 26 ambulance professionals representing all levels of education, during 42 ambulance runs including both day and night shifts and a total of 64 h of observation at a single ambulance station in the North Denmark Region, covering an urban area in the period March to April 2019.

### 2.4. Data Collection

**Figure 1:**
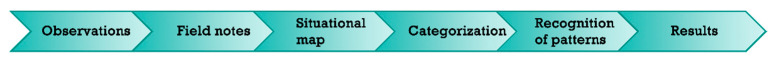
Flowchart of work progress.

The two main authors, Jensen and Ladefoged, observed and followed ambulance professionals during their shifts and accompanied them on ambulance runs to observe the use of the ePMR. Notes from the observations were written down. A template for writing field notes was established with the sections place and action, feelings, and conversations. The two main authors made detailed jottings during observations, which were later written into extended notes. Finally, informal interviews were carried out with the ambulance professionals. (Figure 1).

### 2.5. Analysis

The two main authors carried out the analysis. With inspiration from grounded theory the empirical generated data were analyzed and a situational map was created to analyze the field notes [17]. All data were categorized into main themes and investigated for any potential patterns. We summarized the contents of the patterns into general descriptions regarding the interaction between the ePMR and ambulance professionals as well as how the ePMR affects the ambulance professionals’ work practice. All excerpts that are presented in the Results section come from fieldnotes that were generated in participatory observations in the ambulances. Details are omitted to protect the privacy of the workers.

## 3. Results

**Figure 2:**
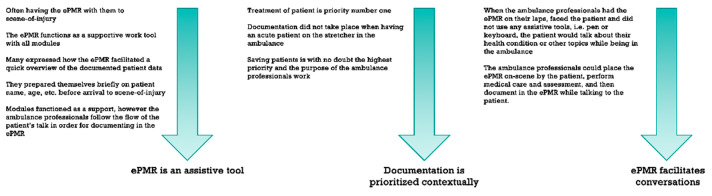
Categorizations of themes into main patterns.

Out of 10 themes, we identified three main patterns that are the basis of the results section, namely that the ePMR is an assistive tool, documentation is prioritized contextually, and ePMR facilitates conversations. (Figure 2).

### 3.1. Ambulance Professionals’ Interaction with ePMR

The ambulance professionals described that the ePMR was not only a new way of documenting, but also an eminent tool. We observed that the ambulance professionals brought the ePMR with them to the scene of injury as well as used it in the ambulance and carried it along into the ED. They reported that the ePMR’s layout helped them to create an overview of data collection and systematically gain a clinical depiction of the patient, which we also observed. Among the ambulance professionals, the ePMR was described as a tool that included a holistic picture of the emergency patient as it facilitated a form of checklist after handover. For instance, an ambulance professional expressed,

*“Whenever I have handed over a patient, I always go through the ePMR, like a checklist, to see if I forgot to type/document anything (in the ePMR)”.*

However, the ambulance professionals emphasized that their “clinical eye” and medical experiences dominated while they collected patient data. One ambulance professional elaborated with an example of when he compiled a patient’s anamnesis. He stressed that the ePMR did not dictate the order of questions, neither the questions to ask—the patient did. He explained that he liked to follow the communicative direction the patient went, as he also felt this could calm the patient in the ambulance. The ambulance professional emphasized that the layout of the ePMR facilitated easy access to each page. He explained that it was fast to jump from one page to another, which made it possible for him to type in data while having an ongoing conversation with the patient. 

The ambulance professionals frequently reported great satisfaction concerning simultaneously sharing data between the ambulance and ED. As one ambulance professional explained:

*“It is much easier to deliver an emergency patient than it was before we had the ePMR. It (ePMR) is a good tool as we now collaborate more actively”.*

Collaboration between EMS, i.e., the ambulances, and the ED was described to have been improved after the implementation of the ePMR. For instance, a camera function in the ePMR is frequently used for severe traffic accidents. An ambulance professional explained that the picture of the scene of injury is shown on a large screen in the trauma room at the ED, and the surgeons use this to both prepare and to estimate the force of impact. Several ambulance professionals also emphasized that the ePMR’s chat function, which provided direct communication with the coordinating nurse in ED, could assist collaboration. As one ambulance professional told us:

*“Back in the days, we experienced that they (the doctors and nurses) threw out the paper records when we came to deliver the patient at the hospital… Now we collaborate—through the ePMR”.*

and

*“In specific situations, you want to communicate messages to the ED in a discrete way. For instance, if the patient is covered with feces”.*

Most of the ambulance professionals reported improved collaboration between ED clinicians and ambulance professionals after implementation of the ePMR. Preparing the ED clinicians through the ePMR for a specific patient arriving in an ambulance was something the ambulance professionals considered valuable in prehospital emergency care.

During the participatory observations, conversations with younger ambulance professionals revealed that whenever the ePMR was malfunctioning it caused them to use a paper medical record and an ambulance professional expressed:

*“It becomes very clear that when the ePMR is not working I use a lot of time documenting (in the paper medical record) and I actually realize that the ePMR is very helpful in my daily work… It is timesaving when it’s (ePMR) working”.*

The ePMR was observed to be an assistive and interactional tool for clinical practice among ambulance professionals. It was found that ambulance professionals were familiar with the ePMR, as they easily and effortlessly documented in the ePMR. We found that the interaction between ePMR and ambulance professionals was influenced by this familiarity, which came into sight as they pointed to specific suggestions for improvements of content in the ePMR. More than one feature of the ePMR was identified as redundant, e.g., the limitation of fewer than 500 words in the note section, double registration of certain data and problems with precision in the drawing tool used for marking the affected areas in burn victims. The ambulance professionals also had the notion and experience that specific documented data in the ePMR were never used in-hospital. The greatest concern expressed by the ambulance professionals within the ePMR features, were restricted access to information on prescribed medicine.

### 3.2. Documentation as Secondary Priority

The ePMR was used contextually, always with the patient’s well-being in focus. Interruptions such as life-threatening situations and short ambulance runs meant the documentation was postponed. Likewise, ambulance professionals emphasized that in acute situations, direct communication with ED per telephone was used, rather than typing in the ePMR. One ambulance professional expressed,

*“It is of great value to be able to call the ED when having a very acute patient—then you are assured that the professionals in the ED gathers the right team within the hospital before we arrive. It is a lot faster to tell the nurse the situation over the phone. The risks of just documenting in the ePMR with a very acute patient are many. It could be a matter of life or death. You have to treat and save the patient! That is your number one priority”.*

However, important samples such as vital signs were observed to be taken in every run, which were automatically transferred from the monitor to the ePMR. In situations that were not life-threatening, we observed documentation to have higher priority. However, ambulance professionals mentioned that distances affected the level of documentation, i.e., shorter distances in an urban area made it difficult for the ambulance professionals to both treat the patient and document simultaneously. When handing over patients in ED to the coordinating nurse, we observed how the ambulance professionals would use information from the ePMR during a verbal handover. In many cases, the ED clinicians had not read the ePMR prior to ambulance arrival. A pattern was observed where ambulance professionals in short phrases described the patient’s condition and conducted treatment to a coordinating nurse at the ED. To this an ambulance professional said,

*“It is a part of our working culture (to verbally hand-over the patient). That is just what we do. This gives us the opportunity to be certain that we communicate the most important information regarding the patient”.*

Only when arriving with patients in critical/life-threatening conditions, we were told that the ePMR had been read beforehand in the ED. However, we observed that the coordinating nurse in the ED gained fast overview using the central ePMR. The ambulance professionals expressed an understanding of unpredictable and busy workflow in the ED, where a verbal handover could help overcome the large patient flow. 

### 3.3. Mobility of the ePMR Invites to Conversation

When ambulance professionals gathered the patient’s history in the ambulance, we observed how they would place the ePMR tablet on their laps and face the patient which created a room for conversation while documenting. The pause that followed when ambulance professionals typed information into the ePMR, seemed to allow room for the patient to impulsively talk about the incident or/and well-being. For instance, a patient started to talk about an upcoming journey with some of the patient’s family members, as the ambulance professional started to type the triage into the ePMR. Another patient first began to describe the anxiety and scare of the traffic accident the patient had just been in, when the ambulance professional started to document basic information into the ePMR. Contrary to the observations, most of the ambulance professionals explained that they generally had the notion of creating distance between them and the patient when using the ePMR and an ambulance professional said:

*“If you are having a conversation and the person you are talking to looks at their phone, you feel like they are not listening… In the same way we create a bigger distance between us and the patient whenever we start typing in the ePMR”.*

However, it was recognized by some ambulance professionals that the ePMR did not completely obstruct the patient relation. For instance, an ambulance professional spoke of the ePMR not being an obstacle to establishing a relationship with the patient:

*“Well yes, it (when typing in the ePMR) can expand the distance between us and the patient, but when you gather information from the patient and concurrently register the data you can actually obtain a fine relation with the patient”.*

Our observations in the ambulances together with the statement above show how the ambulance professionals have awareness of how the ePMR can affect the relationship with the patient, both negatively and positively.

## 4. Discussion

In this study, we found that the ePMR is considered an advantageous work tool, praised by the ambulance professionals, however it cannot stand alone in certain situations, i.e., life threatening situations. Furthermore, the mobility of the ePMR appears to have an effect on conversations between ambulance professionals and patients. 

The ambulance professionals emphasized the support the ePMR provides to their work practices, i.e., the ability to act patient centered with a fast and easy device and communicating with the ED. Our observations confirm this, as the ambulance professionals found utilizing the ePMR a good tool for actively collaborating in a prehospital setting. Moreover, we found that documentation was a secondary priority, and that the ambulance professionals had difficulties completing documentation in the ePMR before arriving at the ED due to short distances. Additionally, it was found that the ePMR cannot stand alone in life-threatening situations. It is evident that these situations call for fast and direct contact with the ED, which the ePMR can provide to some extent. In life-threatening situations, the measured vital signs being transferred in real-time from the monitor to the ePMR, is considered of special importance. 

A previous study concerning how health care work is collaboratively accomplished, have found that EPRs are not just information, but intricately involved in collaborative work and in care processes [2]. We argue that the ePMR can be supportive of collaborative work in life-threatening situations to some extent, as it provides important vital signs in situations where fast information sharing is needed, hence available/shared vital information about emergency patients are considered essential in the collaboration between ambulance professionals and ED, and is confirmed by other studies [18,19,20]. However, the ambulance professionals need to call ED clinicians per telephone in life-threatening situations as their priority is to communicate essential information fast, where documenting in the ePMR would slow the process.

The literature supports our findings concerning how the ambulance professionals are affected by the ePMR in their clinical work practice, i.e., a collaboration between ED and ambulance professionals [2,7,21]. Most of the ambulance professionals reported increased collaboration effort with the ED clinicians following the implementation of the ePMR. This may be explained by the fact that the ED clinicians can gain patient overview in the central ePMR, which they access during handover. This is seemingly because the documented data in the ePMR from the ambulances are used for delegating the patients in the ED and deciding/preparing for further care process in ED. However, the finding of clinical documentation as secondary priority may impede the early decision making regarding care processes in ED, as multiple studies confirm [22,23,24].

We found verbal handover evidently embedded in the ambulance professionals work culture, despite automatic data transfer from the ePMR. Other studies found EMS systems to contain challenges regarding lack in communication between ambulance professionals and hospital clinicians, and that paramedics experienced frustration and a fragmentation in communication when verbally handing over patients in the ED, as nurses were either busy or attended the patient straight away without hearing the handover [7,25]. In our study, we observed that the ambulance professionals acknowledged the unpredictable flow and busyness in ED, and the verbal handovers were facilitated by the presence of a coordinating nurse. The fact that the ePMR is on a mobile tablet computer invites conversations with patients and does not obstruct the ambulance professional’s relationship with the patient. However, it can be questioned if the ambulance professionals listened to the content of what the patient talked about, or if the focus only was on documenting in the ePMR. The ambulance professionals themselves spoke of how documenting in the ePMR would create distance where our observations on the contrary showed that documenting was not an obstruction for the relation with patient. The ambulance professionals’ notion relies on a mindset where it is impossible to be focused on both documenting and listening. However, our observations indicate that they both document and listen, which we will argue to be a habit and great quality of observance embedded in the ambulance professionals work practice. 

A study, performed in-hospital, found that a mobile ‘computer-on-wheels’ in a hospital ward was not used as anticipated because of practical issues, i.e., unreliable network [4]. The study articulates how unstable networks and ‘dead zones’ in scattered areas of the hospital resulted in nurses having to relog on the system and use time on navigating in the record to continue their task. However, most nurses would give up using the electronic device and used paper medical records instead as they were considered more stable and reliable [4].

Furthermore, studies found that mobile documentation systems weakened interpersonal communication between professionals, that the computer seemed less ‘human’ and disturbing while having interpersonal communications, and using tablet based documentation systems created concern among health care professionals in-hospital, as they felt a detached contact with the patient [3,4]. Contrary, in our study the ePMR was identified to be able to lose wireless connection and log the documented information when later regaining connection, which is beneficial when ambulances are in ‘dead zones’. However, in rare cases where the ePMR did not work, e.g., system errors for log-in, the ambulance professionals had to take paper documentation into use. The reason for taking paper documentation into use in our study differs from the previously mentioned study, as the nurses used paper documentation, not due to system error, but because they gave up [4].

### Limitations

The primary limitation of the study is the setting. A single ambulance station in an urban area was included, which may have resulted in observations different from, e.g., rural areas with longer distances to the ED for the ambulance. The ambulance professionals’ awareness is aimed at treating the patient; however, the urban area has an impact on how and when ePMR is taken into use. It is possible that the urban area prescribes the usage of the ePMR and determines the ambulance professionals’ priorities as their awareness are aimed towards the patient and not documenting in the ePMR on short ambulance runs. In addition, the North Denmark Region has used the ePMR since 2006 and has gained intimate knowledge and experience with this tool. Therefore, the experiences of ambulance professionals in this region may differ from those of ambulance professionals from other Danish regions, where the ePMR was first introduced in 2015. Another limitation was that communication between prehospital doctors and ambulance professionals was not included in this study. We thereby lacked insight into this specific collaboration, which could have contributed with a broader perspective to our findings.

We found that the ePMR have had a positive effect on the collaboration between the ambulance professionals and the ED clinicians. This is based upon the observations of the ambulance professionals’ handover at the ED and ambulance professionals’ statements during informal interviews. In the future, it would be relevant to study how the ED clinicians experience the collaboration between them and the ambulance professionals through the ePMR, and how the ePMR has affected the ED clinicians’ work practice.

A major strength of this study is that the two main authors agreed upon a strategy for performing team ethnography before going on the ambulances, hence creating a common strategy for observations and doing field notes. This allowed us to create/gain equal comprehension of the phenomena observed as well as decide focus points when observing. Another strength has been the numerous informal interviews that were carried out during the 64 h of observation. The informal interviews gave valuable insight into knowledge which was embedded in the ambulance professionals’ work practice and otherwise would not have been unfolded.

## 5. Conclusions

This study provides novel information on the ePMR implemented in ambulances, and its effect on ambulance professionals’ work. We found that ambulance professionals have a natural and competent interaction with the ePMR, concerning basic documentation, and collaboration with the ED. The mobility of the ePMR facilitates conversation and affects the patient relationship in a positive way, allowing ambulance professionals to act towards patient centered care. However, the ePMR cannot stand alone when collaborating across the prehospital setting as handovers in the ED are affected by verbal handovers and telephone calls are necessary in life-threatening situations. Thus, the ePMR demonstrated an appropriate use of technology aiding ambulance professionals in their work in the prehospital setting. Use of ePMR in highly acute situations could be of particular interest in future research.

## Data Availability

The data generated and analyzed in this study can be made available from the corresponding author on reasonable request.
